# Social media landscape: a cross-sectional survey of health professionals

**DOI:** 10.1007/s00296-025-06000-4

**Published:** 2025-10-23

**Authors:** Akerke Auanassova, Kanon Jatuworapruk, Manali Sarkar, Marlen Yessirkepov, Maidan Mukhamediyarov, Lisa Traboco, Ashish Goel, Olena Zimba, Vikas Agarwal, Elena Nikiphorou, Latika Gupta, SOMER Study Group, Wilson Bautista-Molano, Christopher Edwards, Jeffery A. Sparks, Carlo Vinicio Caballero-Uribe, Manouk de Hooge, Kim Lauper, Peter Boyd, Ghita Harifi, Russka Shumnalieva, Francis Berenbaum, Dzifa Dey, Peter Kerkhof, Loreto Carmona, Yoshiya Tanaka, Cristiana Sieiro Santos, Tsuneyasu Yoshida, Taanya Talreja, Felix Mühlensiepen, Stefka Stoilova, Mwidimi Ndosi, Chuanhui Xu

**Affiliations:** 1 Department of Education, Research, and Strategic Development, Heart Center Shymkent, Shymkent, Kazakhstan; 2 Department of Internal Diseases, Khoja Akhmet Yassawi International Kazakh-Turkish University, Shymkent, Kazakhstan; 3 Department of Medicine, Faculty of Medicine, Thammasat University, Pathumthani, Thailand; 4 Sir H. N. Reliance Foundation Hospital and Research Centre, Mumbai, Maharashtra India; 5 Center for Life and Health Sciences, National Academy of Sciences Under the President of the Republic of Kazakhstan, Almaty, Kazakhstan; 6 Department of Chemical Disciplines, Biology and Biochemistry, South Kazakhstan Medical Academy, Shymkent, Kazakhstan; 7 Department of Scientific Works, South Kazakhstan Medical Academy, Shymkent, Kazakhstan; 8 Luke’s Medical Center-Global City, Taguig, Philippines; 9 University of the Philippines, Manila, Philippines; 10 Department of Medicine, Dr BR Ambedkar State Institute of Medical Sciences, Mohali, Punjab India; 11 Department of Rheumatology, Immunology and Internal Medicine, University Hospital in Kraków, Kraków, Poland; 12 National Institute of Geriatrics, Rheumatology and Rehabilitation, Warsaw, Poland; 13 Department of Internal Medicine N2, Danylo Halytsky Lviv National Medical University, Lviv, Ukraine; 14 Department of Clinical Immunology and Rheumatology, Sanjay Gandhi Postgraduate Institute of Medical Sciences, Lucknow, India; 15 Centre for Rheumatic Diseases, King’s College London, London, UK; 16 Rheumatology Department, King’s College Hospital, London, UK; 17 Department of Rheumatology, Royal Wolverhampton Hospitals NHS Trust, Wolverhampton, UK; 18 School of Infection, Inflammation and Immunology, College of Medicine and Health, University of Birmingham, Birmingham, UK; 19 Francis Crick Institute, London, UK

**Keywords:** Surveys and questionnaires, Digital media, Medicine, Healthcare workers, Social-media

## Abstract

Social networks are widely used in the healthcare system for education, research, and professional networking. However, its adoption and impact in Central Asia, particularly among medical professionals, remain underexplored. This study assesses social media usage patterns, motivations, and perceived challenges among healthcare professionals in Kazakhstan, with a focus on professional engagement, information-seeking behaviours, and the need for structured training. A cross-sectional online survey was conducted among healthcare professionals in Kazakhstan from November 2022 to January 2023 after extensive pilot-testing by a multi-professional team. The questionnaire comprised multiple-choice and open-ended questions, and Likert scale answers to explore, social media preferences for professional engagement, and future possibilities. Among 147 respondents (M:F ratio = 1:1.17, median age = 32 years), social media adoption was nearly universal (97.96%, n = 144). The primary reasons for usage were knowledge acquisition (81.94%, n = 118), skill development (79.16%, n = 114), and maintaining social connections (68.05%, n = 98). YouTube and Instagram were the most frequently accessed platforms. Despite the perceived professional utility of social media, 75% (n = 108) of respondents felt overwhelmed, and only 65.27% (n = 94) considered it a secure means of communication.69% (n = 99) had never attended training on optimal social media use for professional growth, and 98.61% (n = 142) expressing willingness to participate in future trainings. The social media landscape among healthcare professionals in Kazakhstan reveals nuanced patterns of platform utilisation. The perceived utility of these platforms is tempered by acknowledged challenges, highlighting a critical need for structured guidance and comprehensive professional training frameworks tailored to the unique digital communication environment of Central Asian healthcare settings.

**Supplementary Information:**

The online version contains supplementary material available at 10.1007/s00296-025-06000-4.

## Introduction

In the continuously evolving digital world, social media has transformed into a vibrant space, enabling users to produce, distribute, and interact with various content and experiences [1]. Current studies emphasize the essential importance of digital health literacy, referred to as e-health literacy, as a critical factor influencing how users engage with digital medical technologies [2]. Healthcare professionals can leverage social media to enhance health results, establish a network of colleagues, stay informed about news and advancements, inspire patients, and share health information with the community [3, 4]. Social media has transformed from a platform primarily for entertainment to one increasingly utilized for professional reasons, such as keeping informed about clinical trials, obtaining treatment and dosage details, networking with colleagues, and participating in continuing professional development activities [1].

Social media has transformed professional communication globally, but its adoption among Central Asian medical professionals may differ from worldwide trends. Social, financial, religious, political, cultural, and racial factors can influence the level of engagement with social media platforms in different areas and restrict participation [5]. Variations in language are another significant barrier preventing citizens of these nations from engaging in international scientific discussions [6]. While these platforms can enhance healthcare communication and networking, their impact in Central Asian healthcare settings is less understood [5].

In Kazakhstan and neighbouring countries, local cultural norms, technological infrastructure, and professional practices likely influence social media usage in the medical field. Implementation and effectiveness in Central Asian academic and medical communities may require culturally sensitive approaches. A global study (EMEUNET) examined social media utilization among rheumatology researchers and scientists [7], revealing active engagement for both personal and professional purposes. However, this study may not fully represent the Central Asian context [5].

This cross-sectional study aims to assess the current social media landscape among healthcare medical professionals in Kazakhstan. By understanding the unique challenges and opportunities in this region, we can better tailor strategies for effective social media integration in Central Asian healthcare settings.

Our findings contribute to the development of culturally sensitive strategies that enhance social media implementation and effectiveness in Central Asian academic and healthcare communities, addressing gaps in global studies and providing practical guidance for local healthcare professionals and policymakers.

## Materials & methods

### Study design and ethical approval

Our study employed an online survey adapted from the SoMER study group’s [8] instrument **[**Supplementary Table S1**],** consisting of 30 questions covering demographics, professional background, and social media usage patterns specific to the Central Asian medical community. This was inspired by the original EULAR-EMEUNET survey reported by Nikiphorou E et al. [7], which provided perspectives on how rheumatologists’ across 47 countries use social media (SM). Informed consent was obtained electronically and the institutional ethical approval was obtained from the local ethics committee at South Kazakhstan Medical Academy.

#### Data collection

The survey was first translated from English to Kazakh (forward translation) by two independent bilingual experts with medical backgrounds. Then, a separate bilingual translator, blinded to the original version, performed the back translation into English. Discrepancies between the original and back-translated versions were reviewed and reconciled by a panel consisting of a medical researcher, a language specialist, and a layperson to ensure both conceptual and linguistic equivalence. This procedure was carried out over seven rounds of revisions to enhance wording, eliminate ambiguities, and ensure cultural relevance and clarity for respondents from Kazakhstan. This thorough approach sought to maintain the purpose and significance of the initial questionnaire while ensuring it is completely comprehensible and applicable to the local setting [9].

A preliminary test was carried out with a limited number of participants (n = 10) to consider the transparency and coherence of the adapted questionnaire. Minor wording adjustments were made to ensure cultural relevance; however, no major items were added or removed from the original SoMER survey.

The survey underwent content validation by local medical experts prior to distribution. The assessment focused on the structured content’s relevance, clarity, and comprehensiveness to ensure its appropriateness for the target population. Following validation, the survey was hosted on SurveyMonkey and distributed in English and Kazakh via popular social media platforms in Central Asia, from November 2022 to January 2023, aiming to capture nuanced insights into how regional medical professionals engage with social media. Responses were anonymous and mandatory, with partial submissions discarded. We used convenience sampling, relying on voluntary participation and sharing among professional networks in Kazakhstan and neighbouring countries, without offline advertising or incentives, aligning with common research practices in the region.

#### Data extraction and scoring of survey variables

Based on respondents' answers to the initial survey question about social media usage, respondents were categorized into users and non-users of social media. Non-users of social media answered demographic questions, and reasons for non-use. Users of social media provided inputs on time spent, proficiency, barriers and motivation of social media usage, platform and sites followed and perception of social media. Daily usage of social media for professional use was classified as low (< 1 h/day), average (1–3 h/day) and high (> 3 h /day) [10]. A semi-quantitative approach was employed to assess social media platform understanding (1 = very limited understanding to 5 = very good understanding), perceived professional impact (-2 = highly negative to + 2 = highly positive), and the likelihood of maintaining separate personal and professional social media accounts (1 = never to 5 = always). Platform usage frequency was classified as regular (at least weekly), infrequent (less than weekly), or never (no usage) [11]. To ensure transparency and reliability, we adhered to the Checklist for Reporting Results of Internet E-surveys (CHERRIES) [12].

#### Statistical analysis

Data analysis for this study used IBM SPSS Statistics (version 26). We presented categorical variables as frequencies (percentages) and continuous variables as mean (standard deviation) or medians (interquartile ranges) as appropriate. Logistic regression was performed using the Enter method. Demographic variables, along with those identified as significant in previous literature, were included as predictors. The model was adjusted for respondents’ age.

## Results

### Population characteristics

A total of 303 individuals were recruited to participate in this study. However, participants who either did not fully complete the questionnaire or failed to meet the established inclusion criteria were subsequently excluded from the analysis. 147 complete responses were included in the final analysis (M:F ratio 1:1.17). The median age was 32 years (IQR: 29–35), with. 58.50% answering in Kazakh, reflecting local language preferences. Participants had a median of 7 years of experience (IQR: 3.00–10.00) and were primarily practicing physicians (63.94%), or other healthcapпpиare professionals (46.93%), engaged in clinical settings (77.8%), with some in teaching (35.37%) and research (21.76%) [Table 1].

**Table 1:** Respondent characteristics

Respondent characteristics	N	median (IQR) or n (%)
Age	147	32 (29–35)
Gender F: M	145^#^	1.17
Professional title, n (%)*	147	
Student		15 (10.20)
Practicing physician		94 (63.94)
Non-clinical academic/researcher		12 (8.16)
Clinical academic/researcher		40 (27.21)
Healthcare Professional		69 (46.93)
Administrator		19 (12.95)
Registered nurse practitioner		4 (2.72)
Physiotherapist		11 (7.48)
Occupational health therapist		4 (2.72)
Others		3 (2.04)
Years of experience	147	7 (3.00–10.00)
Job setting*	147	
Clinical work		114 (77.55)
Teaching		52 (35.37)
Research		32 (21.76)
Laboratory work		9 (6.12)
Others		8 (5.44)
In academic role	147	59 (40.13)

*F* Female; *M* Male

^*^Multiple answers

^#^Two respondent identified as other gender or preferred not to disclose their gender.

The study involved healthcare professionals, including rheumatologists, internists, and general practitioners, all aged 25 and above. Participants were required to have completed their medical education and be practising physicians. Individuals currently enrolled in medical programs under the age of 25 were excluded from the study.

### Social media usage and motivation

Kazakhstan had widespread (97.96%) social media adoption among healthcare professionals. Only three respondents said they did not use social media citing reasons of having inadequate time, not suiting their needs and concerns about misinformation on social media. Among professionals who used social media, 43.05% reported challenges in accessing social media due to connectivity issues (30.56%), legal restrictions (16.67%), and hardware scarcity (4.86%).

**Fig. 1:**
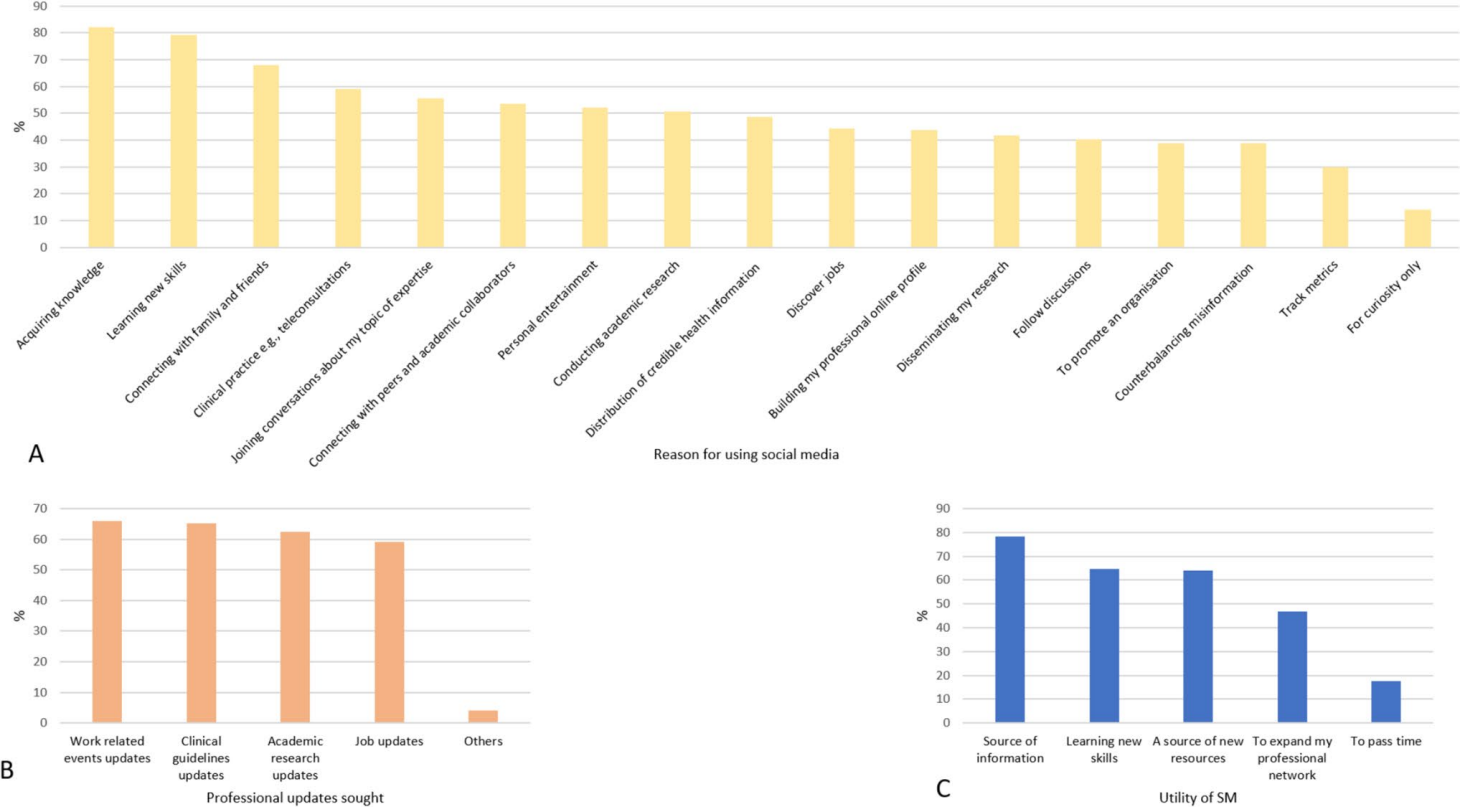
**A** Reasons for using social media **B**. Professional updates sought **C**. Utility of SM*.* *SM* Social media

Daily social media usage was evenly distributed between low (< 1 h/day) and average (1–3 h/day) use, at 47.22% and 45.83%, respectively, while only 6.94% reported high usage (> 3 h/day). The top three reasons for using social media being to acquire knowledge (81.94%), learning new skills (79.16%) and connecting with family and friends (68.05%) [Fig. 1A]. In professional life, social media was used as a source of information (78.23%), learning new skills (64.62%) and receive updates about work (65.97%), clinical guidelines (65.28%) and academic research (62.50%) [Fig. 1B, C].

### Social media platforms used and sites followed

**Fig. 2:**
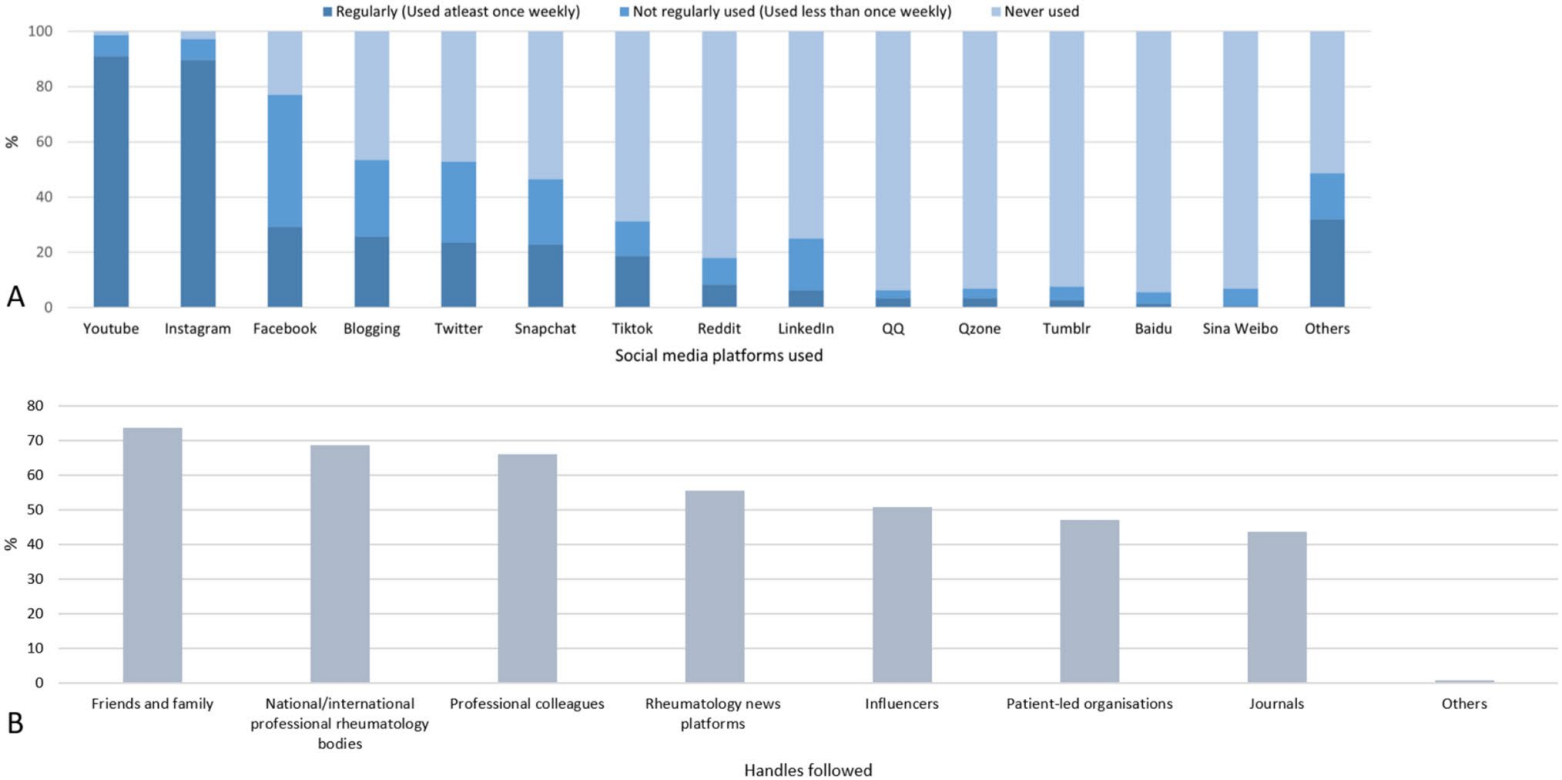
**A** Social media platform used **B**. Handles followed

Platform utilization varied, with YouTube and Instagram used at least once weekly, while Facebook and Twitter were used less regularly. Baidu, Qzone and Tumblr were not used by over 90% of the respondents [Fig. 2A]. Kazakh respondents were more likely to follow friends and families (73.61%) and colleagues (65.97%) than patient led organization (47.22%) and journals (43.75%) [Fig. 2B].

### Perception of social media

Three-fourths of respondents felt overwhelmed by social media, leading 63.89% to contemplate taking a break and 53.47% to have previously done so. Nine out of ten respondents reported acquiring knowledge of educational and professional value and expressed a positive perception of social media’s utility for professional advancement. However, when asked about whether they felt social media was a safe method of communication only thirteen out of twenty agreed [Table 2].

**Table 2:** Perception of social media

Perception	Overall (n = 144)
Impact SM have on professional working life*	4.5 (0.81)
Likelihood of separate personal and professional social media presences*	3.25 (1.46)
Change in perception regarding utility of SM^@^	
Yes, become more positive	92.36
Yes, become more negative	2.78
No, nothing has changed	4.86
Felt overwhelmed by SM^@^	75.00
Contemplated taking a break from SM^@^	63.89
Taken a break from SM^@^	53.47
Acquired knowledge of educational or professional value relating to life sciences/medicine on social media^@^	91.66
Consider SM be a secure way (private, safe from cyber-bullying) of communicating with other people^@^	65.27

^*^Calculated as mean (SD)

@Calculated as percentage of overall sample size

Among Kazakh HCP, perceiving social media as a secure means of communication was associated with increasing age (OR 1.07, 95% CI 1.00–1.14; p = 0.05) and male gender.

### Perceived need for training in communication tools with focus on SM

**Fig. 3:**
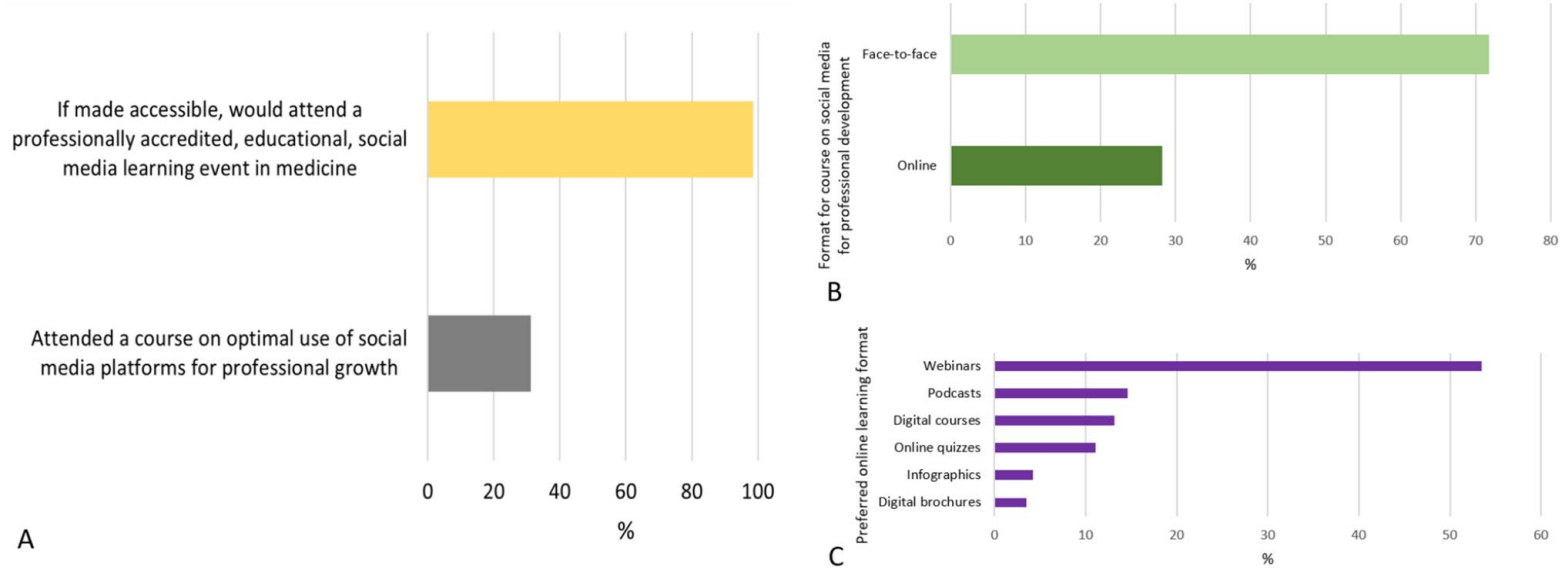
**A** Unmet needs training in digital media tools **B**. Preferred course format for course on social media for professional development **C**. Preferred online learning format for said courses

In Central Asia, previous courses on optimal social media use for professional growth had limited reach, despite high willingness [Fig. 3 A]. Respondents preferred face-to-face sessions over online formats [Fig. 3 B]. Among online formats, webinars (53.47%) were most preferred, followed by podcasts (14.58%), digital courses (13.19%), online quizzes (11.11%), infographics (4.17%), and digital brochures (3.47%) [Fig. 3C].

## Discussion

In this cross-sectional study, we identified the current atmosphere in social networks among healthcare professionals and identified this group of doctors' existing knowledge and practice. Unsurprisingly, all healthcare workers in our study use social networks, aligning with other research [13]. This universal participation may, however, reflect a sampling bias towards digitally engaged professionals. This study showed that most participants use a separate personal and professional presence on social networks. Respondents indicated that they spend considerable time on social media platforms to update clinical and research materials. Healthcare professionals view social networks and online communities as valuable sources for obtaining information to help make informed decisions [14].

Over half of rheumatologists surveyed by EMEUNET preferred Facebook for cooperation and Twitter for journal club meetings though the preferences in Central Asia seemed to be different, with greater focus on Instagram instead. [15–19]. Healthcare professionals in Central Asia seem to widely use YouTube for work-related content. Instagram has become a platform for medical education, with doctors, clinics, and patients sharing their experiences through visual content. Infographics on these platforms have proven particularly effective for medical education and knowledge dissemination. [20]. Research shows an increasing trend among students and professionals to use YouTube for information and education [21].

Despite widespread social media use, our survey revealed significant professional reservations, with seven out of twenty respondents considering platforms unsafe and nearly three-quarters feeling overwhelmed, reflecting deep-seated concerns about information reliability, professional conduct, patient confidentiality, and organisational risks. Our findings align with existing literature, highlighting persistent tensions between digital connectivity and professional boundaries, as well as emerging concerns around the ethical dissemination of health-related content via social media platforms [22, 23]. The survey results suggest that while healthcare professionals are active social media users, they remain critically aware of potential risks, including information unreliability, potential breaches of professional conduct, and patient privacy concerns [24].

Our survey revealed a significant knowledge gap, with 69% of participants never having attended a course on professional social media use. Consistent with other research, approximately 26% of medical professionals identified a need for information education and guidelines governing workplace social network usage, highlighting the potential for structured training to enhance professional confidence and digital literacy. [25]. Low and colleagues found a notable knowledge gap in social media engagement amongst established clinicians, particularly those who qualified 5–10 years ago. This likely reflects the limited emphasis on digital communication during their early careers [26]. As social media becomes increasingly embedded in healthcare, there appears to be a clear need for ongoing professional development in this area. Whilst many clinicians are adept at clinical practice, formal guidance on digital engagement would be beneficial. This might include practical support on maintaining professional boundaries and using these platforms effectively for knowledge exchange [13].

Healthcare professionals with specialist expertise would benefit from thoughtful guidance on social media engagement. Such frameworks might help inform choices about appropriate platforms for different purposes, whether sharing expertise amongst colleagues or engaging with research communities. Whilst social media presents valuable opportunities for professional networking, a measured approach to its integration into clinical practice would be prudent. Different platforms may serve distinct purposes—for instance, professional networks for scholarly discourse versus broader platforms for research recruitment or patient engagement. A gradual, considered adoption of social media tools could enhance professional communication whilst maintaining appropriate boundaries [27]. A thoughtful balance between engagement and discretion would therefore be prudent [28, 29].

To eliminate the identified lack of knowledge in the field of social media use among medical professionals, it is proposed to introduce structured educational programs, include relevant components in the curricula of medical universities, and develop evidence-based guidelines for ethical and practical professional interaction in the digital environment, taking into account the cultural and technological characteristics of Central Asia.

The findings- particularly the widespread use of social media for continuing medical education, professional networking, and patient engagement- are directly applicable to rheumatology, where staying updated with rapidly evolving treatment protocols and interdisciplinary collaboration is critical. Additionally, the research underscores the importance of organised training in digital communication, which could be particularly advantageous in areas such as rheumatology that often overlap with other specialities like cardiology, dermatology, and immunology.

This study provides the first examination of social media usage patterns specifically among healthcare professionals in Central Asia, revealing distinct regional platform preferences and quantifying a substantial training gap in professional social media guidance. These findings highlight universal challenges in professional social media use and suggest the need for internationally coordinated guidelines that can accommodate regional platform preferences within the global rheumatology community.

This study has several limitations that should be acknowledged. First, potential selection bias may have been introduced due to the use of online convenience sampling, which likely attracted digitally engaged individuals and may not accurately represent the broader population of healthcare professionals in Kazakhstan. Second, all data were self-reported, which may carry the risk of reporting bias or subjective interpretation.

Additionally, the limited sample size of this cross-sectional online survey constrains the generalisability of the findings to the wider healthcare community. Future research should aim to recruit a larger and more diverse cohort of medical professionals and include comparative analyses across different regions to enhance the validity and applicability of the results.

## Conclusion

This global e-survey among healthcare professionals’ outlines Instagram and YouTube as the most preferred means of professional engagement in academics from Kazakhstan. Healthcare professionals can effectively use social media for networking and knowledge exchange through structured frameworks, as described in the EULAR Declaration on Social Media Use for Rheumatology Professionals [30], that protect privacy and uphold ethical standards. Digital communication training should be integrated into medical curricula as a key professional skill. While these platforms enhance collaboration, medical organizations need clear strategies to safeguard professional integrity and support early-career researchers in navigating the digital landscape.

## Supplementary Information

Below is the link to the electronic supplementary material.

Supplementary file1 (DOCX 19 KB)

Supplementary file2 (DOCX 22 KB)

## Data Availability

The datasets generated and/or analysed during the current study are not publicly available but are available from the corresponding author upon reasonable request.
