# Supplementary material for: Social media landscape: a cross-sectional survey of health professionals
Source: Rheumatol Int. 2025 Oct 23;45(11):255. doi: 10.1007/s00296-025-06000-4 (PMC12549729; doi:10.1007/s00296-025-06000-4)
Supplement: Supplementary file 1 — Supplementary file1 (DOCX 19 KB) [file 296_2025_6000_MOESM1_ESM.docx]

**Supplementary Material**

**SoMeR Study Group Members and Affiliations:**

1. Wilson Bautista-Molano: 1. Clinical Immunology Group, Universidad Militar Nueva Granada, Bogotá, Colombia. 2. Cellular and Molecular Immunology Group (InmuBo), Universidad El Bosque, Bogotá, Colombia. 3. Department of Rheumatology, Hospital Universitario Fundación Santa Fe de Bogotá, Bogotá, Colombia. ORCID: [0000-0003-0684-9542](https://orcid.org/0000-0003-0684-9542). [wilson.bautista@gmail.com](mailto:wilson.bautista@gmail.com)
2. Christopher Edwards Department of Rheumatology and NIHR Clinical Research Facility, University Hospital Southampton NHS Foundation Trust, Southampton, [UK.cedwards@soton.ac.uk](mailto:UK.cedwards@soton.ac.uk)
3. Jeffery A. Sparks: Division of Rheumatology, Inflammation, and Immunity, Brigham and Women's Hospital, and Harvard Medical School, Boston, Massachusetts, USA. jsparks@bwh.harvard.edu.
4. Carlo Vinicio Caballero-Uribe: Department of Medicine, Hospital Universidad del Norte, Barranquilla, Atlantico, Colombia. carvica@gmail.com. ORCID: 0000-0002-9845-8620
5. Manouk de Hooge: Department of Rheumatology, Ghent University Hospital, Ghent, Belgium. ORCID: [0000-0002-0652-9808](http://orcid.org/0000-0002-0652-9808).  msmdehooge@gmail.com
6. Kim Lauper: Division of Rheumatology, Geneva University Hospital and Faculty of Medicine, University of Geneva, Geneva, Switzerland. ORCID: [0000-0002-4315-9009](https://orcid.org/0000-0002-4315-9009). Kim.Lauper@unige.ch
7. Peter Boyd: Services Support Officer, Arthritis Ireland. 2. Chair, EULAR PARE Committee. ORCID: 0009-0009-4753-8717. [pboyd_2@hotmail.com](mailto:pboyd_2@hotmail.com)
8. Ghita Harifi: Rheumatology Unit, Department of Medicine, Dubai Hospital, Dubai, United Arab Emirates. ghitaharifi@yahoo.fr
9. Russka Shumnalieva: Department of Rheumatology, Clinic of Rheumatology, Medical University-Sofia, Bulgaria. [rshumnalieva@yahoo.com](mailto:rshumnalieva@yahoo.com) . ORCID: 0000-0003-2321-6536
10. Francis Berenbaum:1. Department of Rheumatology, Sorbonne University, Paris, France 2. Department of Rheumatology, Saint-Antoine Hospital, Assistance Publique-Hopitaux de Paris, Paris, France.3. INSERM, Paris, France.[francis.berenbaum@aphp.fr](mailto:francis.berenbaum@aphp.fr).
11. Dzifa Dey:  Department of Medicine and Therapeutics, University of Ghana School of Medicine and Dentistry, College of Health Sciences, Korle-Bu, Accra, Ghana. [drdzifadey@gmail.com](mailto:drdzifadey@gmail.com). ORCID: 0000-0002-3339-5112.
12. Peter Kerkhof: Department of Communication Science, Vrije Universiteit Amsterdam, Amsterdam, Netherlands. [p.kerkhof@vu.nl](mailto:p.kerkhof@vu.nl)
13. Loreto Carmona: Instituto de Salud Musculoesquelética (INMUSC), Madrid, Spain loreto.carmona@inmusc.eu
14. Chuanhui Xu: Department of Rheumatology, Allergy and Immunology at Tan Tock Seng Hospital, Singapore. [xuchuanhui2008@gmail.com](mailto:xuchuanhui2008@gmail.com)
15. Yoshiya Tanaka: The First Department of Internal Medicine, School of Medicine, University of Occupational and Environmental Health, 1-1 Iseigaoka, Kitakyushu, 807-8555, Japan.: tanaka@med.uoeh-u.ac.jp
16. Cristiana Sieiro Santos: Rheumatology Department, Complejo Asistencial Universitario de León, León, Spain. ORCID: 0000-0003-0889-9877. [cristysieirosantos@gmail.com](mailto:cristysieirosantos@gmail.com)
17. Tsuneyasu Yoshida: Department of Rheumatology and Clinical Immunology, Kyoto University Graduate School of Medicine, Kyoto, Japan. [t.yoshida.lym@gmail.com](mailto:t.yoshida.lym@gmail.com)
18. Taanya Talreja: Seth GS Medical College and KEM Hospital, Mumbai, Maharashtra, India. [taanyatalreja@gmail.com](mailto:taanyatalreja@gmail.com)
19. Felix Mühlensiepen: [Medizinische Hochschule Brandenburg Theodor Fontane](https://www.researchgate.net/institution/Medizinische-Hochschule-Brandenburg-Theodor-Fontane?_tp=eyJjb250ZXh0Ijp7ImZpcnN0UGFnZSI6InByb2ZpbGUiLCJwYWdlIjoiaW5zdGl0dXRpb24iLCJwcmV2aW91c1BhZ2UiOiJwcm9maWxlIn19), Neuruppin, Germany. [felix.muehlensiepen@mhb-fontane.de](mailto:felix.muehlensiepen@mhb-fontane.de)
20. Stefka Stoilova:  University Multi-profile Hospital for Active Treatment "Sveti Georgi", Plovdiv, Bulgaria. [Stefka.Stoilova@mu-plovdiv.bg](mailto:Stefka.Stoilova@mu-plovdiv.bg)
21. Mwidimi Ndosi: College of Health, Science and Society, UWE Bristol, UWE Glenside Campus, Blackberry Hill, Bristol [Mwidimi.Ndosi@uwe.ac.uk](mailto:Mwidimi.Ndosi@uwe.ac.uk)
