# Supplementary material for: Social media landscape: a cross-sectional survey of health professionals
Source: Rheumatol Int. 2025 Oct 23;45(11):255. doi: 10.1007/s00296-025-06000-4 (PMC12549729; doi:10.1007/s00296-025-06000-4)
Supplement: Supplementary file 2 — Supplementary file2 (DOCX 22 KB) [file 296_2025_6000_MOESM2_ESM.docx]

**Supplementary table S1: Items in the survey**

| Items | Options |
| --- | --- |
| Do you use social media? | - Yes - No |
| What are your barriers to using social media for professional or educational purposes? | - None - Broadband and connectivity issues - Legal restrictions on access to SoME sites - Lack of hardware to access social media - Other (please specify) |
| What do you use social media for (multiple answers)? | - Acquiring knowledge - Learning new skills - Clinical practice e.g., teleconsultations - Conducting academic research - Disseminating my research - Distribution of credible health information to my patients - Counterbalancing misinformation related to my line of work. - Discover jobs - For curiosity only - Track metrics - Follow discussions - To promote an organisation Personal entertainment - Connecting with family and friends - Connecting with professional peers and potential academic collaborators - Building my professional online profile - Joining conversations about my topic of expertise - Other (please specify) |
| How would you rate your understanding of the use of various social media platforms? | - Very limited understanding - Limited understanding - Average understanding - Above average understanding - Very good understanding - I’m not sure |
| What impact does social media have on your professional working life? | - Highly positive impact - Moderately positive impact - No impact - Moderately negative impact - Highly negative impact |
| Please tick the frequency with which you use the below social media platforms for work related purpose. | SMP-   - Facebook - Twitter - Instagram - Snapchat - LinkedIn - TikTok - QQ - Tumblr - Qzone - Sina Weibo - Baidu Tieba - Reddit - YouTube - WeChat - Other media-sharing sites e.g., Fileshare - Blogging platforms e.g., WordPress, Warwick blogs etc. - Other (please specify)   Respondents were to rate from a scale of-   - Never - Infrequently - At least once monthly - At least once weekly - Atleast once daily |
| What is your average daily use of social media for professional or educational purposes? | - <30 minutes - 30 minutes- 1 hour - 1-3 hours - 3-5 hours - >5 hours |
| Do you have separate personal and professional social media presences? | - Always - Usually - Sometimes - Rarely - Never |
| What work related purposes do you use social media for? (multiple answers) | - Clinical guidelines updates - Academic research updates - Job updates - Work related events updates - Other (please specify) |
| Who do you follow on social media sites? (multiple answers) | - National/international professional bodies - Patient-led organisations Influencers - Professional colleagues - Friends and family - Journals - News platforms (e.g. RheumNow, medicine matters) - Other (please specify) |
| Do you consider social media to be a secure way (private, safe from cyber-bullying) of communicating with other people? | - Yes - No - Other (please specify) |
| Have you ever felt overwhelmed by the content on social media? | - Yes - No |
| Have you ever contemplated taking a break from social media? | - Yes - No |
| Have you taken a break from social media in the last 3 years | - Yes - No |
| Have you ever acquired knowledge of educational or professional value relating to life sciences/medicine on social media? | - Yes - No |
| Which type of online format do you find most helpful in supporting learning for professional development? | - Infographics - Podcasts - Webinars - Digital courses - Digital brochures - Online quizzes - Other (please specify) |
| Have you ever attended a course on optimal use of social media platforms for professional growth? (multiple answers) | - Not yet - Yes - No, I would like to attend one! |
| If made accessible to you, would you attend a professionally accredited, educational, social media learning event in medicine? | - Yes, I would prefer online over a face-to-face event - Yes, but I would prefer a face-to-face event if available - No - I am not sure |
| What are your reasons for not using social media (multiple answers) | - I’m not sure how to use it Concerns about data privacy issues - Concerns about rules on patient engagement - Concerns about cyberbullying - I don’t have time - It isn’t suitable for my needs - I find it distracting - Violation of the Patient–HCP Boundary - Concerns around legal and licencing issues - Concerns around Damage to Professional Image - I am wary of skewed information online inherent to platform algorithms - Concerns about misinformation on social media - I am wary of influencers, financial gains and changing dynamic of medical consumerism - Other (please specify) |
| Has your perception regarding the utility of social media for professional advancement changed in the last 5 years? | - Yes, become more positive - Yes, become more negative - No, nothing has changed |
| In your experience, what are the most important reasons for you to use social media in a work-related manner? | - Source of information - A source of new resources Learning new skills - To expand my professional network - To pass time - Other (please specify) |
| How many years have you been working as a rheumatologist or healthcare professional (including years as fellow-in-training) | - Sliding scale (0-50 years) |
| Which of the following best describes your current job role? (multiple answers) | - Student - Practicing physician - Non-clinical academic/researcher - Clinical academic/researcher - Administrator - Healthcare Professional - Registered Nursing Practitioner - Physiotherapist - Occupational Health Therapist - Other (please specify) |
| Do you currently have an academic role? If so, what is it? | - No - Yes - PhD student - Yes - Postdoc - Yes – Assistant Professor/Associate professor or similar - Yes - Professor - Other (please specify) |
| What do you specialise in? | Text box |
| What is your current job setting? (Multiple answers) | - Clinical work - Teaching - Research - Laboratory work - Other (please specify) |
| What is your age? | Sliding scale |
| What gender do you identify with? | - Male - Female - Do not wish to disclose - Other |
| What is your current country of work? | Drop down menu |
| Would you like to participate in future surveys on social media or get involved with our team to explore the subject? If yes, please provide your email address. Your email address will be strictly kept confidential and for university records only. | Comment box |
